# The sodium–glucose co-transporter 2 inhibitor empagliflozin attenuates cardiac fibrosis and improves ventricular hemodynamics in hypertensive heart failure rats

**DOI:** 10.1186/s12933-019-0849-6

**Published:** 2019-04-01

**Authors:** Hsiang-Chun Lee, Yi-Lin Shiou, Shih-Jie Jhuo, Chia-Yuan Chang, Po-Len Liu, Wun-Jyun Jhuang, Zen-Kong Dai, Wei-Yu Chen, Yun-Fang Chen, An-Sheng Lee

**Affiliations:** 1 Division of Cardiology, Department of Internal Medicine, Kaohsiung Medical University Hospital, Kaohsiung Medical University, Kaohsiung, Taiwan; 2 Department of Internal Medicine, Faculty of Medicine, College of Medicine, Kaohsiung Medical University, Kaohsiung, Taiwan; 3 Center for Lipid Biosciences, Kaohsiung Medical University Hospital, Kaohsiung, Taiwan; 4 Lipid Science and Aging Research Center, Kaohsiung Medical University, Kaohsiung, Taiwan; 5 Department of Mechanical Engineering, National Cheng Kung University, Tainan, Taiwan; 6 Department of Respiratory Therapy, College of Medicine, Kaohsiung Medical University, Kaohsiung, Taiwan; 7 Division of Pediatric Pulmonology and Cardiology, Department of Pediatrics, Kaohsiung Medical University Hospital, Kaohsiung Medical University, Kaohsiung, Taiwan; 8 Graduate Institute of Basic Medical Science, China Medical University, Taichung, Taiwan; 9 Department of Medicine, Mackay Medical College, No.46, Sec. 3, Zhongzheng Rd., Sanzhi Dist., New Taipei City, 25245 Taiwan

**Keywords:** SGLT2 inhibitor, Empagliflozin, Hypertensive, Heart failure, Cardiac fibrosis, Hemodynamics, High-fat, PPARα, ACADM

## Abstract

**Background:**

Sodium glucose co-transporter 2 inhibitor (SGLT2i), a new class of anti-diabetic drugs acting on inhibiting glucose resorption by kidneys, is shown beneficial in reduction of heart failure hospitalization and cardiovascular mortality. The mechanisms remain unclear. We hypothesized that SGLT2i, empagliflozin can improve cardiac hemodynamics in non-diabetic hypertensive heart failure.

**Methods and results:**

The hypertensive heart failure model had been created by feeding spontaneous hypertensive rats (SHR) with high fat diet for 32 weeks (total n = 13). Half SHRs were randomized to be administered with SGLT2i, empagliflozin at 20 mg/kg/day for 12 weeks. After evaluation of electrocardiography and echocardiography, invasive hemodynamic study was performed and followed by blood sample collection and tissue analyses. Empagliflozin exhibited cardiac (improved atrial and ventricular remodeling) and renal protection, while plasma glucose level was not affected. Empagliflozin normalized both end-systolic and end-diastolic volume in SHR, in parallel with parameters in echocardiographic evaluation. Empagliflozin also normalized systolic dysfunction, in terms of the reduced maximal velocity of pressure incline and the slope of end-systolic pressure volume relationship in SHR. In histological analysis, empagliflozin significantly attenuated cardiac fibrosis in both atrial and ventricular tissues. The upregulation of atrial and ventricular expression of PPARα, ACADM, natriuretic peptides (NPPA and NPPB), and TNF-α in SHR, was all restored by treatment of empagliflozin.

**Conclusions:**

Empagliflozin improves hemodynamics in our hypertensive heart failure rat model, associated with renal protection, attenuated cardiac fibrosis, and normalization of HF genes. Our results contribute some understanding of the pleiotropic effects of empagliflozin on improving heart function.

## Introduction

Sodium glucose co-transporter 2 inhibitors (SGLT2i) are a new class of anti-diabetic drugs acting on inhibiting glucose and sodium resorption by the kidneys. Marketed compounds of SGLT2i include dapagliflozin, canagliflozin, empagliflozin, and ertugliflozin. Empagliflozin was approved in August 2014. In addition to efficacy for glycemic control on the HbA1c level, cardiovascular protective benefit from SGLT2i is remarkable, in terms of reduction of hospitalization for heart failure (HF) [1–5], and reduction of cardiovascular death in patients with type 2 diabetes mellitus (DM) [1]. Not only for diabetes patients with systolic heart failure, ongoing clinical trials are performed to evaluate the cardiovascular effects of SGLT2i in pre-diabetes [5, 6]. There are emerging evidences for a diabetic independence that SGLT2i can be beneficial to non-diabetes patients with HF. A recent study showed that empagliflozin reduced myofilament passive stiffness and directly improves diastolic tension of human end-stage HF ventricular trabeculae [7].

Possible mechanisms for the pleiotropic effects of SGLT2i on improving cardiac dysfunction have been suggested [8, 9]. First, lipid oxidation was shown increased to match the decrease in glucose oxidation on SGLT2i, thereby the switch of substrate utilization from carbohydrate to lipids is considered to maintain another energy balance [10]. Second, empagliflozin was also shown to bring significant blood pressure reduction with approximately 3 to 4 mmHg at 12-week [11]. Empagliflozin preserved cardiac microvascular endothelial cell function through suppressed mitochondrial reactive oxygen species production [12]. Empagliflozin also reduces CaMKII activity and CaMKII-dependent SR Ca^2+^ leak which are hallmarks of HF [13]. Although the cardiovascular benefits are striking for SGLT2i, the underlying mechanisms are not fully understood.

In the EMPA-REG OUTCOME trial, empagliflozin is suggested to directly improve hemodynamics since the reduction in HF hospitalization was observed shortly after initiating SGLT2i therapy [1]. Hemodynamics regulation is critical for heart function, particularly for HF. It remains unclear how SGLT2i modulates cardiac hemodynamics in HF. It is also undetermined whether SGLT2i can also be beneficial in non-diabetes heart failure.

We previously reported that high-fat diet induced adverse atrial and ventricular remodeling in old rats with spontaneous hypertension, through upregulation of PPARα, ACADM and TNF-α, and a strong upregulation of α-MHC in atria and ventricles [14]. Using this hypertensive HF rat model, the effects of empagliflozin were investigated, including biochemistry, electrocardiographic parameters, atrial and ventricular remodeling, intra-myocardial fibrosis, and hemodynamic analysis.

## Materials and methods

### Animal models

**Fig. 1:**
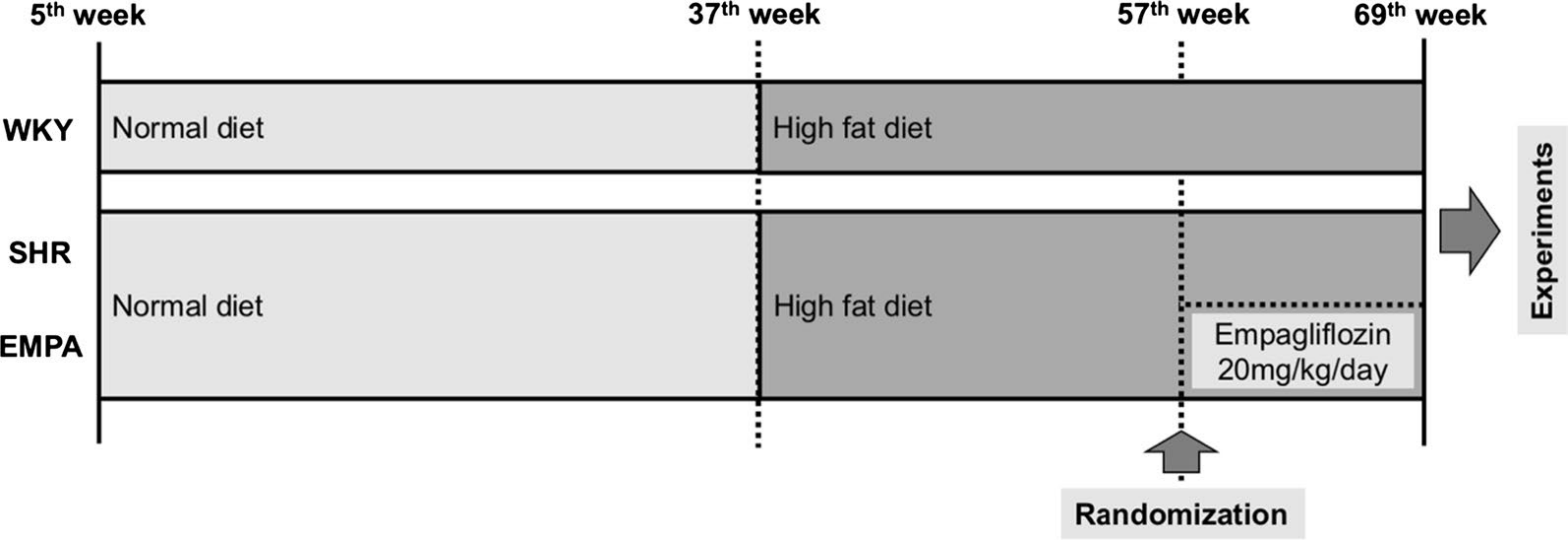
The animal grouping and time line of the experimental protocol

Male, 5-week-old spontaneous hypertensive rats (SHR) and normotensive Wistar–Kyoto (WKY) littermates (controls) were purchased from the National Laboratory Animal Center (Taipei, Taiwan), and housed in a temperature-controlled animal facility (22 °C) with a 12-h light/dark cycle. All animals had been fed with normal diet for 32 weeks, and then high fat diet (HFD, fat content 45% of energy, RESEARCH DIETS, New Brunswick, NJ, cat. D12451) for 20 weeks. Seven SHR rats at 56-week-old were randomly chosen to the EMPA group, which empagliflozin was administered in drinking water with a dose of 20 mg/kg/day, for 12 weeks. Twelve weeks later, electrocardiography, echocardiography, and invasive PV-loop analysis were performed in vivo (Fig. 1). Empagliflozin was kindly provided by the Boehringer Ingelheim Pharma GmbH & Co. KG, Biberach an der Riss, Germany. WKY rats at same age were used as the control group.

### The performance and measurements of echocardiography and electrocardiography

At 69-week-old, recordings were conducted on all groups of animals after being anesthetized with 1.5–2% isoflurane. For the echocardiography, a transducer with a 30 MHz frequency was used. The left ventricular (LV) wall thicknesses [LV posterior wall (LVPW) and interventricular septum (IVS)], and LV chamber dimensions (LVID) at end-diastole were determined from M-mode images. Left atrial size was determined from M-mode at end-systole. The ejection fraction (EF) and LV mass and end-diastole and end-systole volumes (LVEDV and LVESV) were calculated according to Teichholz et al. [15]. For the electrocardiogram (ECG), signal capture was accomplished with platinum electrodes inserted subcutaneously in four limbs and connected to a custom-built ECG amplifier and recorded for about 5 min at 2 MHz (IX-RA-834, iWorx Systems, Dover, New Hampshire). Motion-altered ECG signal and artifacts were discarded before analysis. Recordings were analyzed for intervals with LabChart 5 software (ADInstruments, Dunedin, New Zealand), and QTc intervals were calculated with formula for rats: QTc = QT/(RR/150)1/2 [16].

### Hemodynamic measurements

Rats were anesthetized with intraperitoneal injection of thiopental sodium (60–80 mg/kg ip). Right carotid artery was cut down to insert the microtip 2.0 F Pressure-Volume (PV) catheter (SPR-838, Millar Instruments; Houston, TX). After arterial pressure was recorded, the catheter was advanced to the LV guided by pressure tracing waves as described previously [17]. After stabilization, signals of pressure and volume were continually recorded by using a P–V conductance system (MPVS Ultra, emka TECHNOLOGIES, Paris, France) coupled to a digital converter (ML-870, ADInstruments, Colorado Springs, CO). Hemodynamic parameters were measured under different preloads, which were elicited by transiently compressing the abdominal inferior vena cava.

### Blood sample collection and biochemical measurements

After hemodynamic study, venous blood was drawn from the inferior vena cava into collecting vials, and plasma was prepared and stored at − 20 °C pending further analysis. Plasma samples were used for the measurement by means of specific kits of the following parameters: fructosamine, triglycerides, total cholesterol, low-density lipoprotein cholesterol (LDL-C), high-density-lipoprotein cholesterol (HDL-C), blood urea nitrogen (BUN), creatinine and, aspartate amino-transferase (AST), alanine amino-transferase (ALT) activities, uric acid, and atrial natriuretic peptide (ANP).

### Histology of rat hearts

After venous blood collection, hearts were harvested and processed accordingly for subsequent tissue and molecular analyses. Rat hearts were blotted dry on tissue paper and weighed, then were fixed with 4% paraformaldehyde. Next, they were embedded in paraffin and sections were stained with Masson’s trichrome according to the manufacturer’s protocol as previously described. The whole hearts were scanned at 20-fold magnification on a high-resolution microscope by using TissueFAXS software (TissueGnostics, Vienna, Austria). The fibrosis content was quantified by identifying and counting the number of blue-staining pixels as a percentage of the total left atrial or left ventricular tissue area using ImageJ software.

### Second-harmonic generation microscopy

Collagen deposition in cardiac sections were analyzed by the second-harmonic generation microscopy (SHG). Multiphoton imaging was performed using 10 μm thick acute slices without de-waxing and staining, and acquisitions were taken with a high numerical aperture (NA) objective lens (UPlanSAPo 20×/NA 0.75, OLYMPUS, Japan) which enabled visualization of a major part of the slice. Visualization of micrometer or submicrometer collagen fibers that were heterogeneously distributed in the cardiac tissue at millimeter scale required both a large field of view and a good spatial resolution. The SHG signal was excited by a femtosecond Ti: Sapphire laser (Tsunami, Spectra-Physics, USA) adjusted to wavelength of 800 nm and a fluorescence filter (FF01-390/40-25, Semrock, USA) was specific chosen for SHG detection. We therefore acquired 200 × 200 μm^2^ SHG images with 512 × 512 pixels at pixel scanning rate of 30 kHz and had laser power of 22.4 mW on the specimen.

### Quantitative real-time reverse transcriptase PCR

Small pieces of atrial and ventricular tissue were placed into liquid nitrogen for snap freezing and later stored in − 80 °C freezer until analysis. Four rats in each group were chosen for heart mRNA expression analysis. Total RNA was prepared using TRI Reagent (Sigma-Adrich, St Louis, MO), then reverse transcribed (Invitrogen, Carlsbad, CA). Quantitative real-time RT-PCR was performed using an ABI 7500 real-time system (Applied Biosystems, Foster City, CA) and TaqMan Universal Master Mix II with TaqMan probes (Applied Biosystems, Foster City, CA): PPARα, Rn00566193_m1; ACADM, Rn00566390_m1; TNF-α, Rn01525859_g1; ANP, Rn00664637_g1 and BNP, Rn00580641_m1.

### Data analysis and statistics

Data were expressed as mean ± SD; n indicated the number of rats. One-way ANOVA and Tukey’s multiple comparisons test were used. Statistical significance was considered as a P value ≤ 0.05. Statistical analyses were undertaken using GraphPad Prism software (GraphPad Software; San Diego, CA).

## Results

### The effects of empagliflozin on body weight and biochemistry data

Table 1 showed basic characteristics and biochemistry data among WKY, SHR, and EMPA groups. Compared to normotensive rats (WKY), hypertensive rats (SHR and EMPA) consumed more diet but had lower body weight. Therefore, the principal comparisons are of SHR who received no EMPA treatment and SHR who received EMPA. The EMPA-treated SHR had a significantly lower weight than the non-EMPA treated rats (EMPA 420.9 ± 18.4 g vs SHR 452.0 ± 17.3 g, P = 0.0383). In SHR rats, both systolic and diastolic arterial pressures were higher while compared to WKY rats (114.7 ± 3.8 vs 80.7 ± 4.3 and 87.7 ± 5.3 vs 47.0 ± 5.6 mmHg, respectively, P = 0.002). Empagliflozin treatment significantly improved systolic and diastolic arterial pressures (83.0 ± 2.7 and 45.4 ± 3.6 mmHg, respectively, P = 0.002) when compared to SHR rats. Plasma concentration of glucose, triglyceride, total cholesterol, LDL-C, HDL-C, BUN, creatinine, AST, ALT, uric acid and ANP were listed in Table 1. Compared to WKY, SHRs had lower levels of total cholesterol, HDL-C, LDL-C, triglyceride and fructosamine, and higher level of BUN. EMPA did not significantly affect the fructosamine concentration, which reflects blood sugar level over the preceding 20 days [18]. EMPA was neutral to plasma cholesterol and triglyceride levels in hypertensive rats (Table 1). EMPA caused a significant reduction in creatinine level for hypertensive rats (EMPA 0.47 ± 0.07 vs SHR 0.59 ± 0.10 mg/dL, P = 0.0469).

**Table 1:** The blood pressures, weights, and biochemistry data

	WKY (n = 9)	SHR (n = 7)	EMPA (n = 7)	P value (ANOVA)
Body weight (g)	511.2 ± 26.8	452.0 ± 17.3^a^	420.9 ± 18.4^bc^	< 0.0001
Heart weight (g)	1.92 ± 0.14	2.40 ± 0.43^a^	2.44 ± 0.26^b^	0.0029
Systolic pressure (mmHg)	80.7 ± 4.3	114.7 ± 3.8^a^	83.0 ± 2.7^c^	0.002
Diastolic pressure (mmHg)	47.0 ± 5.6	87.7 ± 5.3^a^	45.4 ± 3.6^$^	0.002

	WKY (n = 5)	SHR (n = 7)	EMPA (n = 7)	P value (ANOVA)
Fructosamine (µmol/L)	254.3 ± 66.9	155.2 ± 44.9^a^	144.6 ± 61.7^b^	0.0027
BUN (mg/dL)	12.9 ± 1.8	21.8 ± 1.6^a^	22.5 ± 4.8^b^	0.0002
Creatinine (mg/dL)	0.56 ± 0.07	0.59 ± 0.10	0.47 ± 0.07^c^	0.0519
AST (IU/L)	167.3 ± 52.5	190.0 ± 40.6	262.5 ± 138.6	0.1919
ALT (IU/L)	74.3 ± 68.4	174.2 ± 89.0	225.5 ± 194.3	0.1927
Uric acid (mg/dL)	1.79 ± 1.09	2.92 ± 0.82	2.39 ± 1.05	0.1731
Triglyceride (mg/dL)	79.8 ± 9.0	59.4 ± 6.7^a^	58.4 ± 7.3^b^	0.0003
Total cholesterol (mg/dL)	120.1 ± 10.0	64.6 ± 6.8^a^	64.6 ± 23.9^b^	< 0.0001
LDL-C (mg/dL)	33.6 ± 4.9	18.7 ± 4.7^a^	17.1 ± 7.2^b^	0.0003
HDL-C (mg/dL)	90.7 ± 7.2	47.5 ± 4.3^a^	49.2 ± 21.2^b^	< 0.0001
ANP (pg/mL)	82.8 ± 18.6	158.8 ± 69.1	93.8 ± 17.5	0.0659

Data are presented as mean ± standard deviation

With Tukey’s multiple comparison test

^a^Comparisons significant for SHR vs WKY

^b^Comparison significant for EMPA vs WKY

^c^Comparison significant for EMPA vs SHR

### Empagliflozin did not change electrocardiographic parameters

**Fig. 2:**
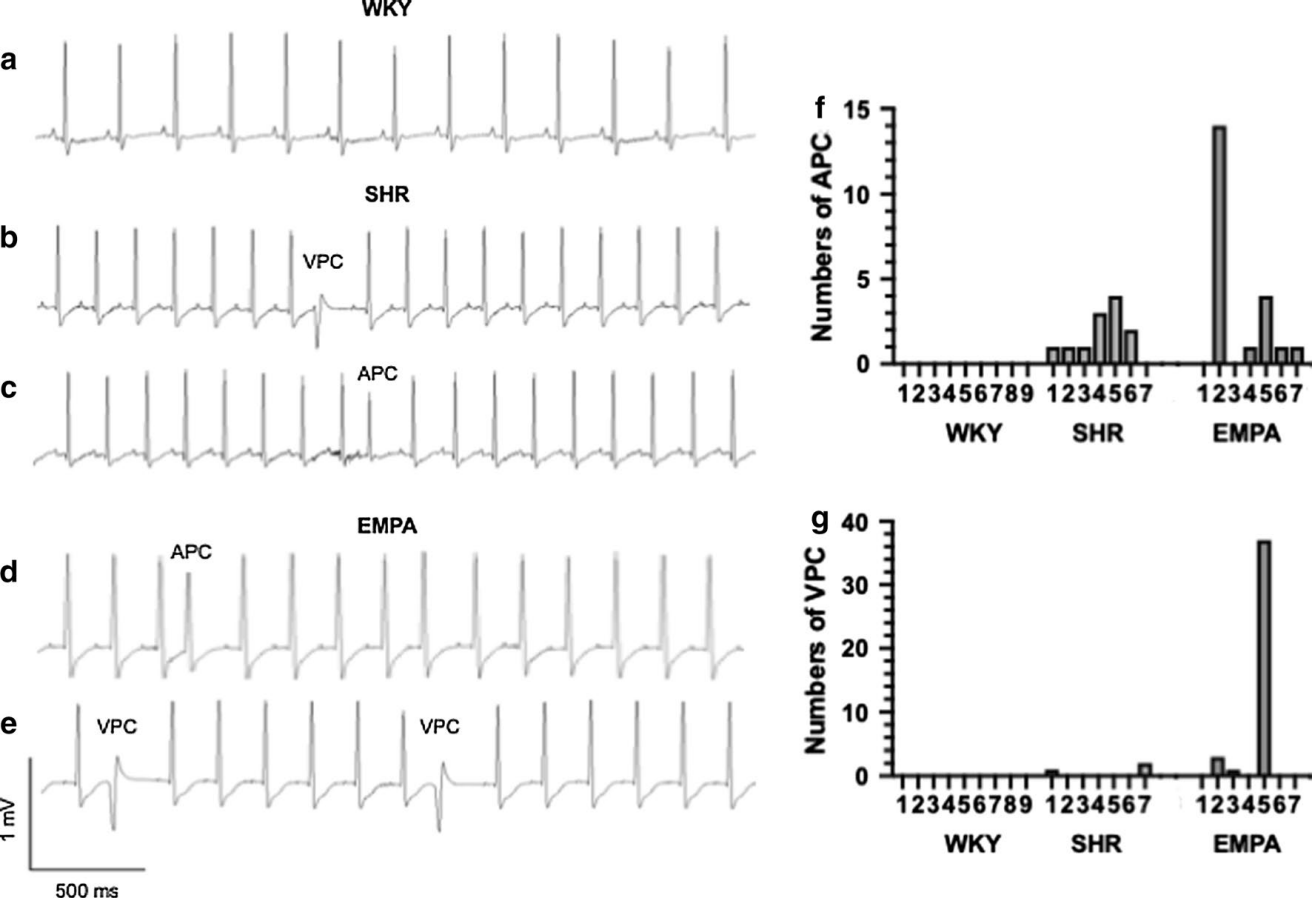
Occurrence of atrial and ventricular premature beats in hypertensive rats. Representative electrocardiographic tracings for normotensive rats (WKY, trace **a**), hypertensive rats (SHR, traces **b**, **c**) and empagliflozin-treated hypertensive rats (EMPA, traces **d**, **e**). Premature beats were specifically labeled as APC for an atrial origin, and VPC for a ventricular origin. The number of APC and VPC for each rat (**f**, **g**) was calculated from 5 min ECG recording

Few atrial and ventricular premature beats (APC and VPC) were observed in only hypertensive rats, with and without empagliflozin treatment (Fig. 2). The number of APC and VPC occurrence in 5 min ECG recording among groups was not significantly different (for APC, ANOVA P = 0.1843; for VPC, ANOVA P = 0.2754). There was also no significant difference among three groups in surface ECG, on parameters of P wave width, PR intervals, QRS width, QT and QTc intervals (Table 2).

**Table 2:** The electrocardiographic parameters

	WKY (n = 9)	SHR (n = 7)	EMPA (n = 7)	P value (ANOVA)
RR (ms)	201.6 ± 26.5	178.7 ± 18.6	193.8 ± 20.4	0.1862
P width (μs)	20.1 ± 1.24	20.8 ± 0.43	19.6 ± 1.7	0.2930
PR (μs)	54.3 ± 4.5	60.3 ± 3.0	59.2 ± 6.4	0.0710
QRS (μs)	30.2 ± 2.3	34.3 ± 5.1	34.5 ± 5.2	0.0923
QT (μs)	109.4 ± 27.0	91.7 ± 7.9	98.3 ± 8.4	0.2028
QTc (μs)	161.2 ± 20.3	154.6 ± 12.2	152.7 ± 9.4	0.5212

Data are presented as mean ± standard deviation

With Tukey’s multiple comparison test

### Empagliflozin improved left atrium size but did not improve left ventricular mass and LV EF

**Fig. 3:**
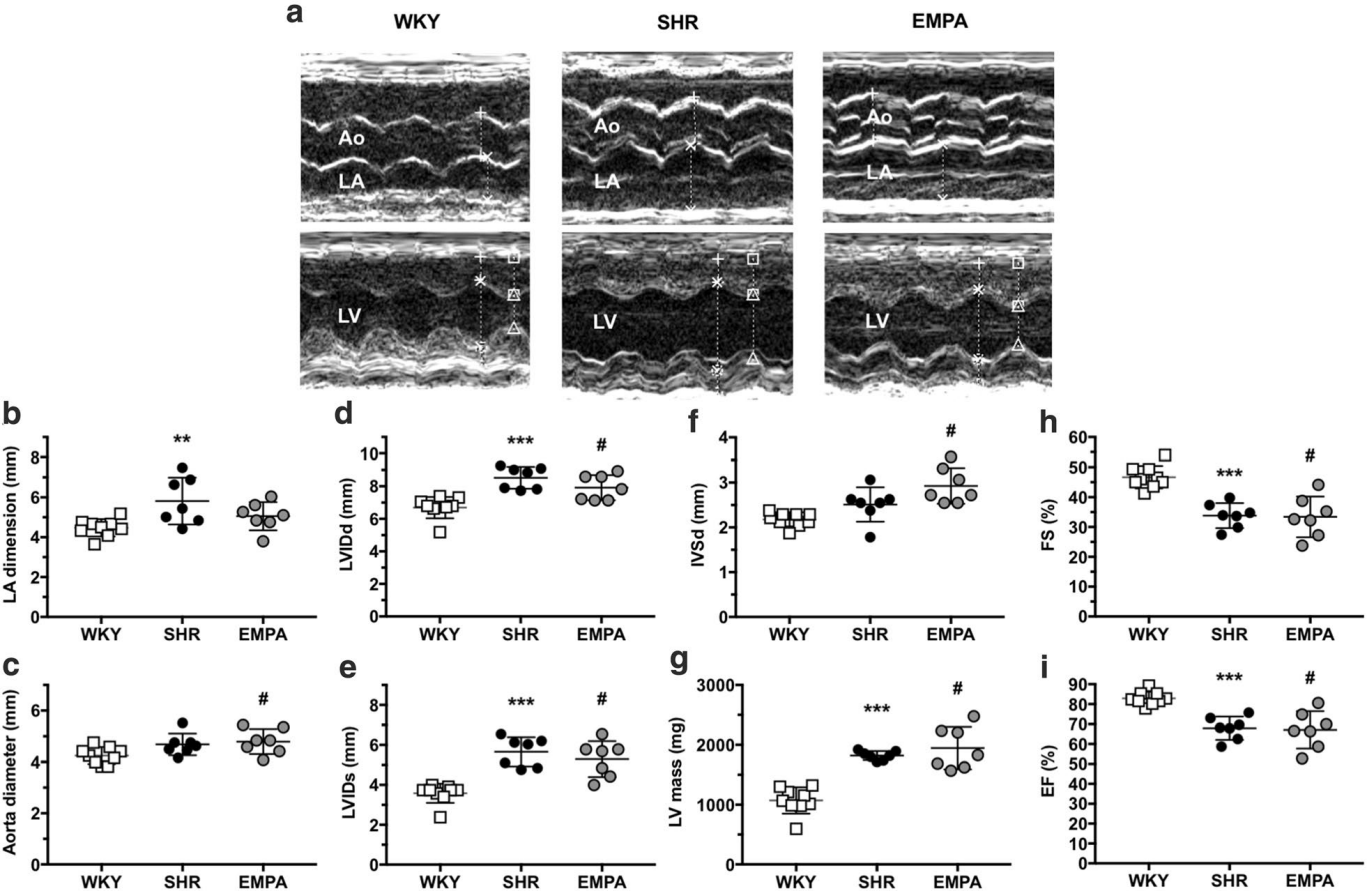
Echocardiographic measurements in normotensive (WKY) and hypertensive rats without (SHR) and with administration of empagliflozin (EMPA). **a** Representative M-mode tracings for aortic root diameter (Ao), left atrial dimension (LA), and left ventricle (LV) measurements of rats in three groups (total n = 9 for WKY, n = 7 for SHR, n = 7 for EMPA). **b**–**i** Plots of echocardiographic measurement results for LA dimension, aorta diameter, end-diastole and end-systole dimensions of left ventricle (LVIDd and LVIDs), end-systole septum thickness (IVSd), LV mass, fraction shortening (FS), and ejection fraction (EF) of LV. **P < 0.01 vs WKY, ***P < 0.001 vs WKY, ^#^P < 0.05 vs WKY

Being fed with the same high-fat diet, cardiac systolic dysfunction was noted in hypertensive rats (LV EF, SHR 67.9 ± 5.8% vs WKY 2.9 ± 3.4%, P = 0.0004). Empagliflozin did not significantly improve the LV EF (67.1 ± 9.4%) (Fig. 3). For left atrial dimension, SHR (6.32 ± 0.77 mm) was significantly larger than WKY (4.52 ± 0.44 mm) (P = 0.009). EMPA improved hypertension-induced left atrial dilatation (5.06 ± 0.71 mm). For left ventricle remodeling, empagliflozin did not significantly improve the chamber size (LVIDd, SHR 9.04 ± 0.21 vs EMPA 7.91 ± 0.77 mm, P = 0.2657), the septal thickness (SHR 2.24 ± 0.4 vs EMPA 2.93 ± 0.40 mm, P = 0.06), and the LV mass (SHR 1821.3 ± 74.8 vs WKY 1946.4 ± 355.5 mg, P = 0.6069).

### Empagliflozin significantly improved high-fat diet-induced cardiac dysfunction in SHR rats

**Fig. 4:**
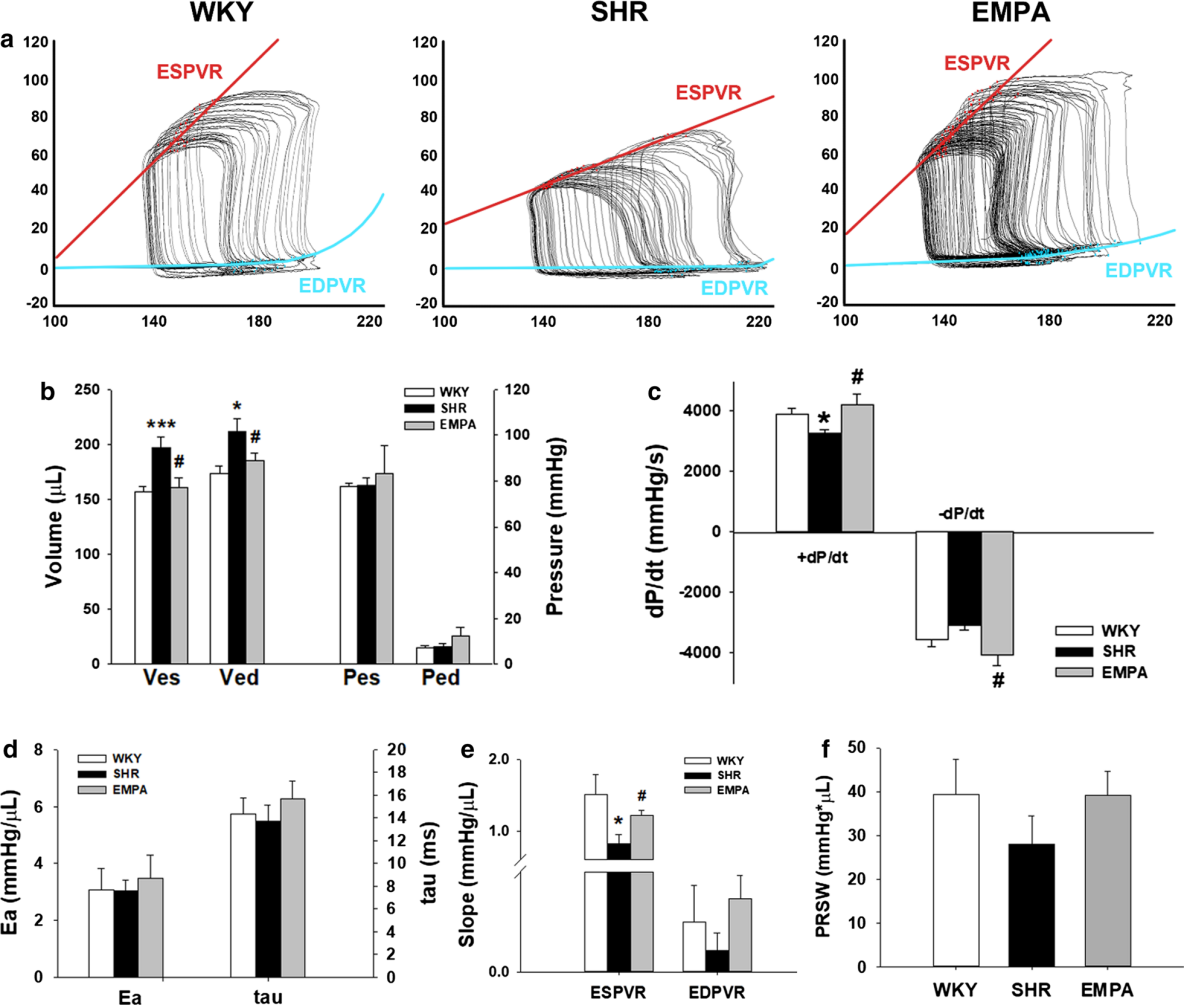
Empagliflozin restored HFD-induced systolic dysfunction in SHR rats. **a** Representative P–V loops at different preloads in WKY, SHR, and EMPA rats. **b** Comparison of the mean end-systolic volume (Ves), end-diastolic volume (Ved), end-systolic pressure (Pes), and end-diastolic pressure (Ped) in the 3 rat models. **c** Comparison of the maximal velocity of pressure rise (+ dP/dt) and fall (− dP/dt) in the 3 rat models. **d** Comparison of the mean arterial elastance. (Ea) and the time constant of isovolumic pressure decay (tau) in the 3 rat models. **e** Mean slopes of the ESPVR and the EDPVR are shown for the 3 rat models. For the quantitative analyses, n = 5 per group. *P < 0.05, ***P < 0.01 vs WKY rats; ^#^P < 0.05 vs SHR rats

To establish the role of empagliflozin in HFD-induced cardiac dysfunction, we treated SHR rats with empagliflozin and then examined their cardiac function by performing P–V loop analysis. Figure 4a shows representative results of the P–V loop analyses with different preloads in WKY, SHR, and SHR fed with empagliflozin. Both end-systolic (Ves) and end-diastolic volume (Ved) were higher in SHR, and were reversed by empagliflozin (Fig. 4b). Likewise, maximal velocity of pressure incline (+ dP/dt) and decline (− dP/dt) were reduced in SHR, and were recovered by empagliflozin treatment (Fig. 4c). We also found that both arterial elastance (Ea) and exponential decay of the ventricular pressure during isovolumic relaxation (tau) were not significantly different among three groups (Fig. 4d). To determine the preload-independent parameters, the abdominal inferior vena cava were transiently compressed during the PV loop analysis. Although the slopes of the end-diastolic pressure volume relationship (EDPVR) curves were not significantly different among groups, the slope of the end-systolic pressure volume relationship (ESPVR) was blunted in SHR (Fig. 4e). Treatment of empagliflozin significantly reversed this effect. On the other hand, beneficial effect was also shown for the preload-recruitable stroke work (PRSW) in empagliflozin group. It reversed SHR-induced decrease of PRSW, but there was no statistical significance (Fig. 4f).

### Empagliflozin significantly attenuated cardiac fibrosis in both LA and LV

**Fig. 5:**
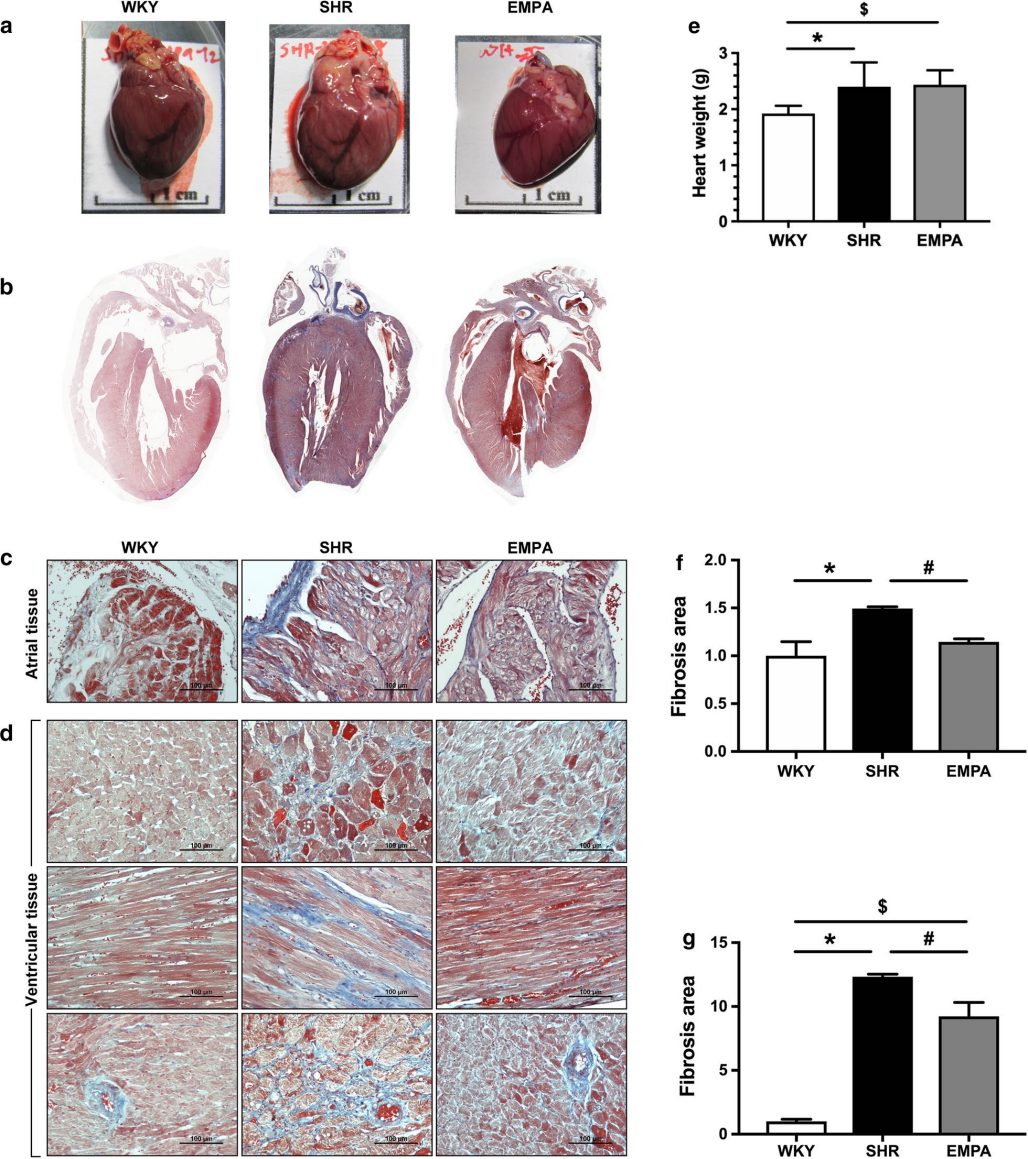
Attenuated fibrosis in empagliflozin-treated atria and ventricles. Gross hearts (**a**), histological sections (**b**), Masson trichrome staining of atrial tissue (**c**) and ventricular tissue (shown in cross, longitudinal sections, and peri-vascular sections) (**d**) of high-fat-fed wild type (WKY), spontaneous hypertensive rats (SHR), and empagliflozin-treated SHR (EMPA). **e** The numerical data of the heart weight was shown. The quantified fibrosis area shows significant increase in SHR compared to WKY and significant attenuation by EMPA, in both atrial (**f** *P = 0.0045; ^#^P < 0.0001) and ventricular tissue (**g** *P < 0.0001; ^#^P = 0.0084; ^$^P = 0.0002)

Although empagliflozin did not reduce the heart weight (SHR 2.40 ± 0.43 g vs EMPA 2.44 ± 0.26 g, P = 0.97) (Fig. 5a), it significantly attenuated cardiac fibrosis (Fig. 5b). Hypertension substantially increased intracardiac fibrosis in left ventricular tissue (SHR was approximately 12.3-fold of WKY, P < 0.0001, Fig. 5c, e), and in atrial tissue with a milder degree (SHR was approximately 1.5-fold of WKY, P < 0.0001, Fig. 5d, f). With 12-week empagliflozin treatment, intra-cardiac fibrosis was significantly attenuated in left ventricles (EMPA was approximately 9.2-folds of WKY, with a 25.2% reduction when compared with SHR, P = 0.0003), and in left atria (EMPA was approximately 1.1-folds of WKY, with a 23.3% reduction when compared with SHR, P = 0.0003).

### Second-harmonic generation microscopy

**Fig. 6:**
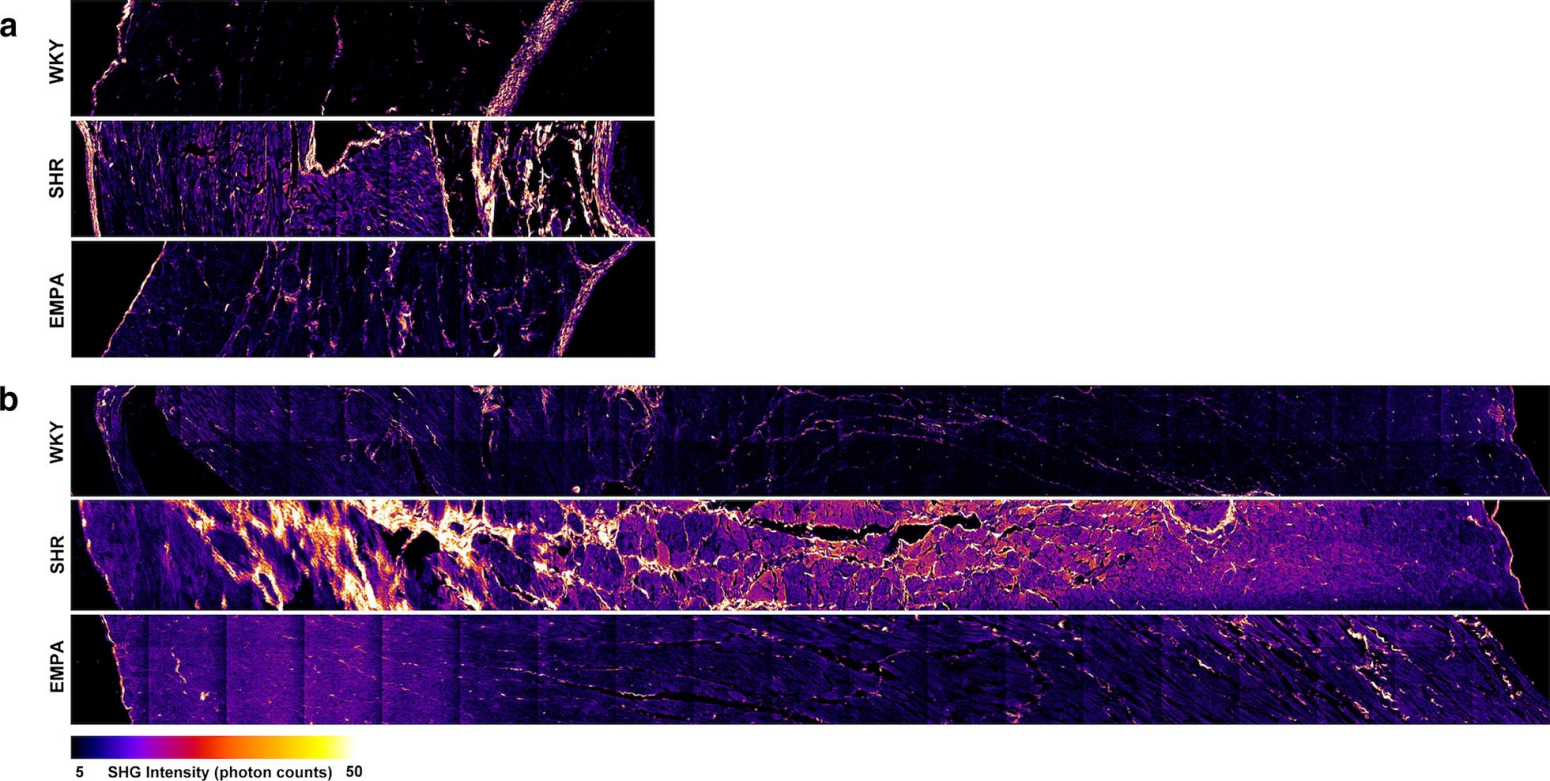
Empagliflozin attenuates hypertension-induced collagen deposition in cardiac fibrosis of left atrial and left ventricular. Collagen fiber deposition and arrangement was demonstrated by second-harmonic generation (SHG) microscopy for left atrial (**a**) and left ventricular (**b**) tissue of normo-tensive rats (WKY), spontaneous hypertensive rats (SHR), and empagliflozin-treated SHR (EMPA). All scans are transmural and show the whole myocardial wall (from left to right: endocardium or luminal surface to epicardium or pericardium). Collagen deposition is demonstrated with high SHG intensity

To determine the effects of empagliflozin on collagen deposition in SHR-induced cardiac fibrosis, SHG microscopy (Fig. 6) was utilized. The results showed that high-fat diet along with hypertension induced massive collagen deposition in heart tissue of left atrium (Fig. 6a) and left ventricle (Fig. 6b). The collagen deposition in heart tissue of normo-tensive WKY rats on high fat diets was sparse. Empagliflozin treatment significantly attenuated hypertension-induced collagen deposition.

### Empagliflozin modulates expression of genes related to fatty acid metabolism

**Fig. 7:**
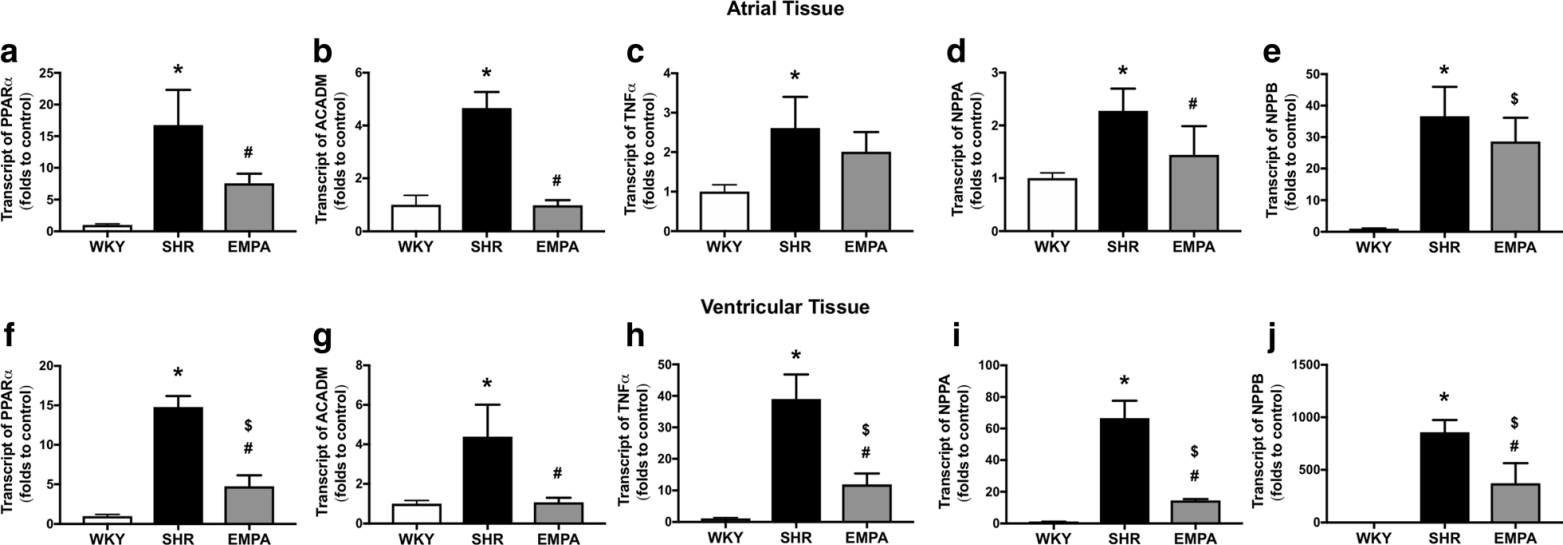
Transcriptional modulation by empagliflozin on genes related to fatty acid metabolism (PPARα and ACADM) and natriuretic peptides (NPPA and NPPB), and TNF-α. The transcriptional expression of genes in the atrial tissue (**a**–**e**) and in the ventricular tissue (**f**–**j**) of control rats (WKY), spontaneous hypertensive rats (SHR), and empagliflozin-treated SHR (EMPA) (n = 4 for each group). Statistical significance is labeled as *for SHR vs WKY; ^#^for EMPA vs SHR; ^$^for EMPA vs WKY

We included two genes that are related to fatty acid metabolism in the real-time PCR experiment. PPARα is a nuclear receptor that is activated by long chain fatty acids to regulate transcriptional activation of genes of key enzymes in fatty acid uptake and oxygenation [19]. The other gene is ACADM that is essential for making an enzyme called medium-chain acyl-CoA dehydrogenase (MCAD) for fatty acid oxidation [20]. Compared to WKY, we observed a significant increase in PPARα transcriptional expression in the SHR atrial (16.76 ± 5.55-folds to WKY; *P = 0.0002) and SHR ventricular tissue (14.79 ± 1.40-folds to WKY; *P < 0.0001) (Fig. 7a, f). The increased PPARα expression was significantly attenuated by empagliflozin for both atria and ventricles (atrial, EMPA 7.54 ± 1.53-folds to control, ^#^P = 0.0089 vs SHR; ventricular, EMPA 4.76 ± 1.39-folds to control, ^#^P < 0.0001 vs SHR). For ACADM transcriptional expression, it was also significantly increased in SHR atrial (4.66 ± 0.61-folds to WKY; *P *< 0.0001) and SHR ventricular tissue (4.39 ± 1.62-folds to WKY; *P *= 0.0018) (Fig. 7b, g). Empagliflozin abolished the increased ACADM in atrial and ventricular tissue of SHR (atrial EMPA 0.98 ± 0.19-folds to control, ^#^P < 0.0001 vs SHR, P = 0.9985 vs WKY; ventricular EMPA 1.073 ± 0.23-folds to control; ^#^P = 0.0021 vs SHR, P = 0.9935 vs WKY).

### Increased TNF-α expression in SHR was attenuated by empagliflozin

Cardiac TNF-α over-expression can induce apoptosis, progressive chamber dilation, and contractile dysfunction [21]. Compared to the WKY control, the transcriptional expression of TNF-α was increased in SHR atrial (2.61 ± 0.79-folds to WKY; *P = 0.0064) and was remarkably upregulated in SHR ventricular tissue (39.01 ± 7.82-folds to WKY; *P < 0.0001) (Fig. 7c, h). Empagliflozin significantly improved upregulation of TNF-α in ventricular tissue of SHR (EMPA, 11.91 ± 3.42-folds to WKY, ^#^*P *< 0.0001 vs SHR, *P *= 0.0316 vs WKY). The effect of empagliflozin on atrial TNF-α was similar to the ventricle without a statistical significance (atrial EMPA, 2.01 ± 0.50-folds to WKY, P = 0.3149 vs SHR, P = 0.0681 vs WKY).

### The upregulation of NPPA and NPPB in atria and ventricles of SHR was attenuated by empagliflozin

The natriuretic peptides (atrial natriuretic factor, ANP and brain natriuretic peptide, BNP) are encoded by NPPA and NPPB genes and both affect the control of extracellular fluid volume and electrolyte homeostasis. NPPA and NPPB are downregulated in adult ventricular tissue and can increase predominantly in condition of heart failure and cardiac hypertrophy [22]. In SHR, both NPPA and NPPB were upregulated in the atrial (NPPA, 2.28 ± 0.42-folds to WKY, *P = 0.0039; NPPB, 36.66 ± 9.32-folds to WKY, *P = 0.0001) and in the ventricular tissue (NPPA, 66.59 ± 11.05-folds to WKY, *P = < 0.0001; NPPB, 858.1 ± 115.7-folds to WKY, *P < 0.0001) (Fig. 7d, e and i, j). The upregulation was most phenomenal with NPPB for the ventricular tissue. The treatment of empagliflozin was shown significantly attenuated both NPPA and NPPB upregulation in ventricular tissue (EMPA, NPPA, 14.54 ± 0.85-folds to WKY, ^#^P < 0.0001 vs SHR; EMPA NPPB, 372.4 ± 190.4-folds to WKY, ^#^*P *= 0.0012 vs SHR) (Fig. 7i, j). For the atria, empagliflozin also significantly attenuated NPPA (EMPA, 1.44 ± 0.55-folds to WKY, ^#^*P *= 0.0395 vs SHR) but the attenuation of NPPB gene was non-significant (EMPA, 28.6 ± 7.54-folds to WKY, *P *= 0.2759 vs SHR) (Fig. 7d, e).

## Discussions

The beneficial effects of empagliflozin shown in this study include: (1) reduced systemic blood pressure, (2) improved renal function, (3) improvement in left atrial dilatation, (4) attenuated intra-cardiac fibrosis, (5) improved incline and decline dP/dt of left ventricle, and (6) modulated genes related to fatty acid metabolism (PPARα and ACADM) and restored upregulated genes in HF.

Like findings in clinical trials [1, 11], empagliflozin reduced blood pressure in our hypertensive rats. Unlikely, in a pre-diabetes, hypertensive metabolic syndrome rat model, empagliflozin did not change blood pressure [23]. The major difference was duration of hypertension, which was 20-week for their SHR/NDmcr-cp (+/+) rats and 67-week for this study. Mechanisms for this SGLT2i effect is suggested as reduction of plasma volume on the basis of natriuretic and osmotic effects, resulting preload and blood pressure reduction.

Myocardial systolic dysfunction was shown in 8-month-old SHR rats fed with Western-type diet [24]. Therefore, we used 37-week-old SHR rats fed with high-fat diet for this study to examine whether empagliflozin could restore systolic dysfunction of high-fat-diet hypertensive rats. To our knowledge, the present study is the first to demonstrate that empagliflozin could attenuate cardiac fibrosis in a hypertensive heart failure model. Increased expression of profibrotic/prohypertrophic proteins, serum/glucocorticoid regulated kinase 1 (SGK1) and the epithelial sodium channel (ENaC) in insulin resistant female diabetic db/db mice were shown improved by EMPA and associated with reduced interstitial fibrosis [25]. The benefit on attenuation of fibrosis from SGLT2i is possibly another drug class effect. For dapagliflozin, the beneficial effect on myofibroblast infiltration and cardiac fibrosis was shown at the remote zone at day 28 after myocardial infarction of normotensive Wistar rats [26]. In addition to the heart, empagliflozin also exerts anti-fibrotic effect on experimental diabetes nephropathy [27]. A favorable alteration in intra-renal hemodynamics on the SGLT2i is suggested [28]. It has also been hypothesized that in diabetes, obese-related diastolic heart failure, the synthesis of leptin leads to sodium retention and plasma volume expansion along with increased cardiac and renal fibrosis [29]. The exact mechanisms of fibrosis attenuation in hypertensive heart disease require more experiments and researches to determine.

Another SGLT2i, dapagliflozin significantly improved LV EF in a HF model that was creased by coronary artery ligation/reperfusion on high-fat diet-induced pre-diabetic Wistar rats. The biological mechanisms on cardioprotection of 4-week dapagliflozin were suggested from a combination of modulation of mitochondrial DRP1, caspase 3, and lipid peroxidation [30]. The LV EF was not significantly improved by 12-week empagliflozin in our hypertensive heart failure model. This discrepancy may be explained by the different disease model (acute myocardial infarction versus chronic hypertensive heart disease), experimental design (administration before the disease versus months after the chronic condition) and animal age (~ 5 months vs ~ 17 months). In this study, both maximal velocity of pressure incline and slope of ESPVR were restored in EMPA group. Although it did not reach statistical significance, the decrease of preload-independent stroke work in SHR group was reversed by 12-week treatment of empagliflozin. The lusitropic defect is a classical indicator of cardiac dysfunction accompanying type 2 DM [31], and empagliflozin was reported to improve diastolic function in terms of improved LV relaxation and compliance in pressure–volume relationship analysis without changing systolic function in a genetic model of type 2 DM [32]. In our study, although the slope of EDPVR was smaller in SHR group and empagliflozin could recover, it did not reach significant difference. The diastolic parameters tau was also comparable among three groups, indicating that diastolic function in all groups of animals were similar.

The effect of empagliflozin on plasma glucose had been examined in non-diabetes human subjects. Empagliflozin can reduce the threshold for glucose spillage into urine to a level below the fasting plasma glucose concentration (~ 30 to 40 mg/dL) [33]. As a result, individuals can still have glycosuria associated with increased sodium excretion in urine after taking empagliflozin, with the maximum inhibition of SGLT2 up to 2 weeks after administration. Approximate 30% urinary glucose is re-absorbed by SGLT1. Unlike individuals with DM, fasting plasma glucose remained unchanged for individuals without DM [33]. Consistently, empagliflozin did not affect plasma glucose level in our non-diabetes hypertensive rats.

There were three hypothesis responding to the diabetes-independent cardiovascular protective effects of empagliflozin: diuretic [34], fuel [35] and thrifty substrate hypothesis [36]. There is increase in hematocrit with initial empagliflozin use, but a robust effect on mortality would not be based on oxygen-delivery dynamics at hemoglobin concentration within the normal range [37]. The cardiovascular protection is suggested to be another drug class effect [3]. Potential mechanisms of cardiac protection with SGLT2i include renin-angiotensin system inhibition following decreased total body sodium content, reduced myocardial cytoplasmic sodium and calcium load, anti-apoptotic, anti-inflammatory, anti-oxidant effects, rescue of diabetes myocardial microvascular injuries, and reduced sympathetic overactivity, inactivation of endoplasmic reticulum stress [6, 12, 38–42]. In this study, empagliflozin restored upregulation of PPARα and ACADM in SHR fed with high fat diet, reflecting a shift toward beneficial metabolic utilization in lipids oxidation. Another novel finding is that empagliflozin restored upregulated TNF-α and activated natriuretic peptides in the hypertensive heart failure rats. These findings may partially explain the beneficial effect in the heart performance. Coinciding with this manuscript preparation there were more recent reports regarding the evaluation of the SGLT2i cardiac protection. Our results still contribute some understanding of the pleiotropic effects of empagliflozin on improving heart performance in a diabetic-independent condition.

The absence of data on the effects of EMPA on myocardial redox aspects, apoptosis, metabolism, intramyocardial sodium and calcium regulation, and insulin sensitivity, is a limitation of this study. In addition, the animals were not housed in individual cages to collect urine that allowed to collect information about glucosuria, albuminuria, and urinary sodium excretion. The lack of a diuretic control group, with an anti-aldosteronic agent, is another study limitation [43–45].

## Conclusions

Empagliflozin exerted beneficial effects on systemic blood pressure, renal function, and ameliorated left atrial dilatation, intra-cardiac fibrosis, contraction and relaxation dysfunction in hypertensive heart failure rats. The potential application beyond type 2 DM for SGLT2i may include hypertension heart failure.

## Abbreviations

ANP: atrial natriuretic peptide
ALT: alanine amino-transferase
AST: aspartate amino-transferase
BUN: blood urea nitrogen
DM: diabetes mellitus
+dP/dt: maximal velocity of pressure incline
-dP/dt: maximal velocity of pressure decline
EMPA: empagliflozin
Ea: arterial elastance
ECG: electrocardiogram
EDPVR: end-diastolic pressure volume relationship
EF: ejection fraction
ESPVR: end-systolic pressure volume relationship
HDL-C: high-density-lipoprotein cholesterol
HFD: high-fat diet
IVS: interventricular septum
LA: left atrium
LDL-C: low-density-lipoprotein cholesterol
LV: left ventricle
LVEDV: LV end-diastole volume
LVESV: LV end-systole volume
LVIDd: left ventricle internal dimension at end-diastole
LVPW: LV posterior wall
P-V: pressure–volume
SGLT2i: sodium–glucose co-transporter 2 inhibitor
SHG: second-harmonic generation microscopy
SHR: spontaneous hypertensive rat
Tau: isovolumic relaxation
WKY: Wistar–Kyoto rat
Ves: end-systolic volume
Ved: end-diastolic volume
